# Prophylaxis of the First Dentition

**Published:** 1911-07-15

**Authors:** N. S. Hoff


### PROPHYLAXIS OF THE FIRST DENTITION.

BY N. S. HOFF, D.D.S.

At what period prophylactic ministrations of any kind should be attempted in the history of the first or deciduous dentition has for many years been a debatable question and is still an unsettled problem. At least no universal agreement will enable us to dogmatize about it in any restricted sense. It becomes therefore necessary to substantiate all innovations by sound logic and documentary evidence in statistical form if practicable. We can not take the space here to present the published results of the examinations made of the mouths of school children showing in most cases a deplorable condition of their teeth, and we have no statistics of consequence showing the conditions prevailing in children from 2 to 5 years—the period which we shall try to cover in this paper,—as these children are not yet in school. Fortunately in the mouths of well cared for children of these ages there is not so extensive carious encroachment as we usually find during the school ages from 6 to 14 in the same class of children.

We meet many cases of extensive destruction of the deciduous teeth; some traceable to faulty calcification or irregular positions, some to severe and prolonged illness and the use of corrosive drugs and consequent insufficient nutrition, and still others, and perhaps the larger proportion, due to lack of cleanliness and especially from lack of proper use. We have already referred to the physiological process of tooth calcification and the manner in which defective results are liable to occur, and will here only make such references as may seem necessary to make more clear the application of remedial agents. We have also shown that preventive treatment should be kept in mind during the entire period of tooth calcification and that it should be more necessary and active as the teeth emerge from the gums and begin to take up their normal function. It would seem that if the teeth were perfectly developed and erupted into normal positions they should take up their function and have a permanent endurance and preserve their integrity as long as they serve a useful purpose in the natural economy. The fact is that they are seldom perfectly developed and in normal position, and besides they are exposed to unusual vicissitudes with none of the protection afforded other organs of the body, hence the greater necessity for artificial protection which, to be most effective, should be instituted at the earliest practicable period.

**Malocclusion of the Deciduous Teeth.** As the first teeth are developed at a time when the maxillae are most plastic and no other organs are present to complicate their development it would seem that they should always take normal positions, and they generally do so. From lack of development of the maxillary bones or alveolar processes they are sometimes crowded and forced into torsal relation, and because of the short cusps they may be shifted into either mesial or distal relations. It may also happen that delayed eruption, caused by failure of the remains of the dental follicle or tooth crypt to absorb at the proper time that some tooth finds its eruptive path difficult or slow of penetration, consequently the adjoining teeth erupting tip towards each other and close the space so that the slowly erupting tooth finds it difficult to secure its proper position in the arch. Such conditions should be noted and corrected at once by such simple procedures as should not cause pain or suffering to the child. If the teeth are erupting into crowded arches the jaws must be stimulated to greater development. This will seldom be in the nature of active and persistent mechanical force, but can often be accomplished by physiological stimulation through systematic massage applied persistently by the nurse or by the little patient himself. If any particular tooth is over-delayed in erupting it may be desirable to determine the cause by means of a skiagram. If the tooth is properly directed and only bound down by overlying fibrous tissue it should be surgically freed and encouraged to emerge; if deflected and its eruption likely to be long delayed some mechanical appliance should be introduced to preserve the space. Small bands on adjoining teeth with a wire soldered between them can be easily made and cemented to place for the youngest patients. If the arches are much crowded it will be wise to begin some method of expansion before the time of replacement of the deciduous by the permanent teeth. This is rather a hazardous procedure as premature eruption of the permanent teeth may be induced or severe inflammation result from the irritation of the alveolar process, which at this time is undergoing active physiologic changes because of the disappearing deciduous and developing permanent teeth. If the crowding is in the lower jaw and the anterior teeth a simple and effective method of expansion is to place a soft german silver wire from cuspid to cuspid resting each end in a small pit drilled in the mesio-labial angle of the lingual surfaces. This wire can be slightly lengthened by pinching it occasionally with a pair of the Angle pliers made for this purpose. It will be surprising how much widening can be done in this way and with little discomfort to the patient. There will also be noticed a considerable widening in the molar region. If the widening is only needed in the molar region for the incoming bicuspids the "Jackson Crib" appliance can be effectively used, care being taken to have the appliance well fitted and with only slight expansion at each adjustment. Various schemes of wedging the teeth apart with cotton tape or fibre ligatures and rubber wedges gaining arch enlargement have been used, but all such methods are objectionable as they become unsanitary if used long enough to produce permanent results and they cause considerable gum irritation and pain to the patient. The use of the Angle arches is not advisable because of the age of the patients, as they annoy the patient very much and are liable to produce excessive results. If the teeth are decayed on their approximal surfaces they are liable to come together resulting in narrowing of the arches. To prevent this the cavities should be cut through to the masticating surfaces and filled with gutta-percha so that in chewing the gutta-percha would wedge the teeth apart by its elasticity. If the cavities are large and in two adjacent surfaces a flat strip of metal should be fitted from one cavity across the inter-proximal space to the other cavity to serve as a kind of floor and prevent the gutta-percha from crowding onto the gum excessively. Whatever is attempted in the way of securing good occlusion and alignment of the deciduous teeth, it should always be remembered that the child is not only very sensitive to pain and can not endure shocks to its nervous system from excessive operations of any sort. The tissues involved are also exceedingly sensitive and grave injuries may result from much interference.

**Caries of the Deciduous Teeth.** These teeth are as liable to disease as the adult teeth, and they probably require as much rescue treatment considering the period of their existence. It is not uncommon to find them decayed within a few months after eruption. Very few children shed the last of their deciduous teeth without having had more or less extensive caries in some of them. We are sometimes inclined to think the deciduous teeth inferior structurally and thus attempt to explain why caries attacks them so readily. Dr. Black maintains that the deciduous teeth are generally well calcified and not different essentially from the permanent teeth in respect to their histologic structure. The deciduous calcification takes place under most favorable conditions along with other rapidly forming osseous development, and there are fewer disease or accidental causes to interrupt normal calcification than obtains in the period of calcification of the permanent teeth. We should therfore expect good tooth organization, although it is not formed as slowly nor from the more mature cells of the permanent tooth follicle.

The carious defects in the deciduous teeth appear most commonly in the proximal surfaces of the upper incisors and the upper and lower molars, and secondly in the fissures of the molars and upper cuspids, and then in the lower incisors. The reason for the proximal caries is undoubtedly due to the crowding of food into the inter-proximal spaces, because the deciduous teeth are not held firmly in the alveolus and separate easily under the force of mastication, permitting food to pass into the interdental space where the broad flat surfaces of the molar teeth retain it until fermentation takes place, because the little ones are not subjected to a proper mouth toilet for its removal after eating. The crowns are kept cleaner, because the fissures are not usually deep and the constant chewing by the child keeps these surfaces fairly free from deposits. The lower incisors are kept clean by the sub-lingual saliva, and their form is such as to make them practically self-cleansing with the aid of the tongue and lips.

**Prophylaxis Treatment.** This treatment should be instituted at the earliest period at which it may be done without injury to the growing roots. At two years the teeth should all be erupted but the entire root development is not completed until about the third year. It is not desirable however to wait until the roots are fully calcified to institute some form of prophylaxis. At the age of two years the crowns are formed and have emerged from the gums and there is sufficient root development and alveolar support to permit the application of sufficient force if properly applied to make all the treatment required. Previous to this about all that may be safely done is to prescribe a suitable mouth toilet that may be applied by the nurse. The teeth should be brushed daily with a suitable brush, not too stiff, and some of the more agreeable tooth pastes or powders used. A satisfactory tooth powder may be made by combining equal parts, by volume, of Prepared Chalk and Calcined Magnesium with a little sugar to make it more palatable and a few drops of wintergreen oil to flavor. If a saponaceous effect is desired add ten per cent powdered palm oil soap.

The consistent use of a mouth toilet, as suggested, should not only keep the crowns of the teeth clean and free from carious attacks, but it should stimulate development of the bones and tissues of the jaw and alveolus, because of the massaging influence on the trophic functions. It will also react on the deep seated organs which are building the secondary teeth.

**Filling Deciduous Teeth.** Filling the deciduous teeth can not be strictly called prophylaxis treatment so far as these teeth are concerned but in a very important sense it is one of the most effective preventive treatments. The retention of the deciduous teeth in form and integrity until the time for their displacement by the permanent teeth is of the greatest prophylactic importance, as it insures proper maxillary development for the permanent teeth and insures proper alignment and occlusion. It also provides the developing child with adequate organs of mastication at the time when he needs all the aids possible for digestion and nutrition. The influence of normally developed teeth and jaws have a great influence in developing the other bones of the face, particularly those associated with the air passages of the nose. It has been clearly shown that faulty development, and even diseases involving the nasal orifices and the air cells of the face, are usually associated with lack of development of the maxillary bones and crowded arches. The probabilities are that these conditions would never have existed if the deciduous teeth had been kept intact until time for their natural loss. Hence the great importance of using every means possible to preserve these first teeth until the permanent teeth are about to supplant them normally.

But for their own best function they should not only be kept in place but in the best possible form to accomplish their work most easily.

At the age of three years it will be practicable to institute a thorough regimen of professional oral prophylaxis treatments. By this we mean that every surface of every tooth crown exposed should be carefully freed and polished of all stains, membraneous attachments, and other extraneous deposits leaving no favoring focus for fermentation and its detrimental influence. With little children this should not all be done at the first sitting but it should be thoroughly done, however many sittings may be required. The better plan is to do only a few of the anterior teeth first and so get the patients confidence and willing cooperation. The polishing should be done with wood points in a hand porte polisher and very fine pumice mixed with dilute phenol sodique. It sometimes happens that there is a deposit of green stain near the gum margins and the enamel beneath is roughened or pitted, making it difficult to remove the stain. In such cases the engine with wood points may be used if care is taken not to injure the gum festoon. In case the enamel is deeply pitted, it may be necessary to dress it down with a fine stone. This should be followed by careful and several times repeated polishings to insure a smooth and hard surface. In some cases the stains are so persistently attached that some chemical reagent needs to be employed. Acids or other drugs which attack the lime salts should not be used. The alkalys are preferable. An application of Iodine which is allowed to stand a few minutes and is then polished away with the phenol-sodique and pumice is usually all that is necessary. Incipient caries on flat surfaces may be dressed down with a small stone in the engine and carefully polished. These pits or cavities may also be treated with nitrate of silver in positions that are not visible.

The polishing of proximal surfaces particularly at the contact points is not practicable with the wood points. For this purpose the broad waxed silk floss answers admirably. Care must be exercised in using it not to allow it to work down against the gum tissue as it is capable of doing much harm to the gums. The crowns of all the deciduous teeth are larger than the roots and there is a distinct contraction at the cervical portion, consequently the polishing tape is very liable to drop into this constriction and cause great injury to the gum. It is the contact points that furnish the conditions favorable to tooth decalcifying reagents and these points should be most carefully polished. If this were carefully done every thirty days and the patients were kept under a good personal mouth toilet regimen, there would not be much occasion for filling the deciduous teeth.

After three years of age caries is liable to appear and the teeth should then receive frequent and most careful inspections. If fillings are indicated they should be made of such materials as can be inserted most readily and with as little pain and discomfort to the patient as possible. The question of durability is not of sufficient importance to justify the use of gold, amalgam or inlays. In locations where no other material is specifically indicated the cements will meet most requirements. In the fissure cavities of the molar teeth the oxyphosphate cements will serve excellently in all incipient or medium sized cavities. In some cases we have found it excellent practice to anticipate caries in the molar fissures and fill the fissures with either oxyphosphate of zinc or oxyphosphate of copper. If practicable place the rubber dam, or secure complete dryness as nearly as is practicable by any other means, and flood the fissures to be treated with some strong sterilizing drug, such as a one to five hundred solution of mercuric chloride in Hydrogen Dioxide, or a ten per cent solution of formaldehyde in alcohol, and work it into every part of the fissure with a very finely pointed probe for three to five minutes. Then thoroughly dessicate with a small stream of high pressure air, not hot, but warm enough to secure complete dryness. Then mix the cement rather thin and work it into the fissures with fine points, using only enough to secure protection and retention and not enough to make occlusion impracticable or uncomfortable. If the hydraulic cements are used, the operation need not be very prolonged and the little patients will not resent it or become irritable. If the cavities involve a considerable part of the occlusal surface and have penetrated the dentine to a considerable extent, fillings are indicated which will not only protect the tooth but wear well under the forces of mastication. In most cases the cements will serve the purpose. But in some cases it will be necessary to place a veneer of amalgam over the cement. This can be done by filling the cavity nearly full with cement and while it is still unset introduce a finishing layer of soft amalgam. If circumstances will permit, the cement can be allowed to set and suitable undercuts made for retention and the balance of the filling be made with amalgam. It is also good practice to fill a part of the cavity with a rather thin mix of cement, and then incorporate a soft mix of amalgam with some of the cement and fill the remainder of the cavity with the combination of cement and amalgam. This makes a durable filling without the objection sometimes made of having the amalgam in contact with the tooth tissue, which it tends to make brittle.

The proximal cavities present the most difficulties. They are many times developed early and the teeth are liable to become seriously affected before the cavities are discovered. They usually develop at the proximal contact points and if seen early the teeth will need to be separated by some slow process of wedging which will secure the needed space without causing much soreness or pain. Cotton tape, or string knotted and tightly tied between the teeth will answer.

In the anterior teeth, if the cavities are small and the rubber dam can be placed so as to keep the cavities dry for a considerable time, the silicate cements will answer very well. If the teeth can't be kept dry long enough for the silicate cement to properly set under dry conditions, it will be better to use the oxyphosphates or gutta-percha. The objection to the oxyphosphate cement is that it dissolves in the oral secretions and by friction so that the contact point is lost, and there is no force in the contact and occlusion of the teeth to keep the arches growing. If the cavities are large there is no other material that can safely be used except the oxyphosphates and silicates, and because of the possibility of injuring the pulps by the strong free acid in silicate cements, there is no alternative but to use the oxyphosphates in the incisal teeth. Proximal cavities of the molars and the distal sides of the cuspids require careful handling and they require most careful supervision, as the preservation of the contact points of these teeth take such an important part in the proper eruption of the secondary teeth. The materials used in filling these teeth should possess insolubility and permanence for at least the time the teeth are expected to remain in the arches. Amalgam has been much used and has some of the desired qualities, but it is so liable to flow away from the cavity margins and crowd onto the gum tissues at the cervical borders that it produces more or less unsanitary conditions unless the fillings are carefully smoothed and polished after they have set and at least twice a year as long as they are doing service. If these teeth have to be filled in the third or fourth year as sometimes happens they will be expected to preserve the tooth for from six to eight years, which is a long life for an amalgam filling in a proximal surface. It is therefore evident that since this is probably the most durable of the practicable materials for this purpose that we should not permit these cavities to develop if prevention is practicable. Obviously the time when filling becomes necessary should be deferred as much as possible. The amalgam and cement mentioned above can be used to good purpose in these cavities, also gutta-percha when there are two cavities in adjacent teeth that can be joined together in one filling. This is rather an unsanitary procedure but it secures expansion.

